# Antidiarrheal Action of *Bacillus subtilis* CU1 CNCM I-2745 and *Lactobacillus plantarum* CNCM I-4547 in Mice

**DOI:** 10.3389/fmicb.2018.01537

**Published:** 2018-07-10

**Authors:** Maria C. Urdaci, Marie Lefevre, Guylene Lafforgue, Christel Cartier, Bertrand Rodriguez, Jean Fioramonti

**Affiliations:** ^1^Microbiology Laboratory, UMR 5248, Bordeaux Sciences Agro, University of Bordeaux, Gradignan, France; ^2^Lesaffre Human Care, Lesaffre Group, Marcq-en-Baroeul, France; ^3^Neuro-Gastroenterology and Nutrition Unit, INRA, Toulouse, France; ^4^Toxalim (Research Centre in Food Toxicology), Université de Toulouse, INRA, ENVT, INP-Purpan, UPS, Toulouse, France

**Keywords:** diarrhea, probiotics, *Bacillus*, castor oil, LPS, CFTR, NHE3, TLR4

## Abstract

Preventive actions of probiotics as antidiarrheal agents are well documented, but their mechanisms are poorly understood. Two selected probiotics, *Bacillus subtilis* CU1 and *Lactobacillus plantarum* CNCM I-4547, were tested in mouse experimental models of diarrhea and the possible mechanisms of action were investigated. Diarrhea was induced in mice by oral castor oil administration or by i.v. injection of lipopolysaccharide (LPS) of *Salmonella enteritis*. The antidiarrheal drug loperamide was used as control. Fecal water excretion was quantified for 2 h and paracellular permeability and electrical parameters of the colon were assessed in Ussing chambers. The expression of colonic exchangers or channels and of Toll-like receptor 4 (TLR4) was assessed by immunohistochemistry. Prophylactic treatment with *B. subtilis* CU1 or with *L. plantarum* CNCM I-4547 reduced LPS-induced diarrhea. The reduction of water excretion was in the same range as those induced by loperamide. In the castor oil model, this effect was only observed with *B. subtilis* CU1. The two probiotic treatments abolished the increase in paracellular permeability induced by LPS, but not by castor oil. However, only *L. plantarum* CNCM I-4547 treatment decreased the colonic expression of TLR-4. After *B. subtilis* CU1, colonic expression of cystic fibrosis transmembrane conductance regulator (CFTR) was reduced and that of Na^+^/H^+^ exchanger 3 (NHE3) increased. *B. subtilis* CU1 may increase the capacity of the colon to absorb excess of water in diarrheic conditions by acting on CFTR and NHE3 expression. The two probiotics strains showed an impact on diarrhea through limitation of water excretion that may involve paracellular permeability or electrolyte transport for *L. plantarum* CNCM I-4547 and *B. subtilis* CU1 respectively.

## Introduction

Diarrhea is one of the most common clinical signs of gastrointestinal disease and can be defined by increased stool frequency, liquidity, or volume. According to the (World Health Organization [WHO], 2013) millions of cases of diarrhea are diagnosed per year in both developed and developing countries. Diarrhea is a condition of altered intestinal water and electrolyte transport and is usually a symptom of an infection in the intestinal tract, which can be caused by a variety of bacterial, viral and parasitic organisms or by the use of antibiotics (AAD) (Sweetser, 2012; World Health Organization [WHO], 2013). Moreover, secretory diarrhea can be induced by some laxatives and a broad range of drugs (e.g., antidepressants, cardiac drugs) (Sweetser, 2012).

Apart from the use of oral rehydration, loperamide is usually the first line treatment in self and non-self-therapy; however, this anti-motility drug can cause adverse effects (Casburn-Jones and Farthing, 2004). For some infectious and traveler’s diarrhea, antibiotics can be used but generally their use is controversial. Therefore, the search for new antidiarrheal strategies becomes an important focus.

Evidence has grown to support the efficacy of probiotics in the management of gastrointestinal disorders many of which are associated with dysregulated fluid and electrolyte transport (Gogineni et al., 2013). Probiotics have been proposed as preventive agents or as complementary therapy for treating diarrhea. Available meta-analyses and randomized, controlled studies using different probiotic preparations revealed that some strains can reduce the duration of diarrhea, shorten the initial phase of watery stools, and decrease the length of hospital stay (Sazawal et al., 2006; Avadhani and Miley, 2011). *Lactobacillus, Bifidobacterium*, and *Saccharomyces* are the most commonly used probiotic strains in the prevention and treatment of diarrhea, but other microorganisms have also been used (Szajewska et al., 2001; Isolauri, 2003). In addition, the efficacy of probiotics as antidiarrheal agents has been reported in traveler’s diarrhea (McFarland, 2007), rotavirus-induced diarrhea (Grandy et al., 2010; Ahmadi et al., 2015) or diarrhea-predominant irritable bowel syndrome (Camilleri, 2013). The ESPGHAN/ESPID guidelines (Guarino et al., 2014) recommended the use of three probiotics (*Lactobacillus rhamnosus* GG, *Lactobacillus reuteri* DSM 17938 and *Saccharomyces boulardii*) in the treatment of children with acute infectious diarrhea as an adjunct to rehydration therapy. Concerning antibiotic-associated diarrhea (AAD) in children, the ESPGHAN (Szajewska et al., 2016) recommended the use of *L. rhamnosus* GG and *S. boulardii*.

Evidence exists for the antidiarrheal properties of *Bacillus* probiotics for human and animal use. Endospore formers such as *B. subtilis* are interesting because their spores are resistant to acidic pH and stable for long periods in probiotic preparations, even in tropical countries (Cutting, 2011) where the incidence of diarrhea is high.

Recently *B. subtilis* 3, tested in a randomized, double-blind, placebo-controlled clinical trial during antibiotic therapy, significantly decreased the incidence of human AAD and adverse effects related to the use of antibiotics (Horosheva et al., 2014).

The mechanisms of antidiarrheal effect of probiotics are poorly understood and few studies showed the action of probiotics on ion-transporters. *In vitro* models point out direct effects induced by probiotics on intestinal cells that can influence the intestinal ion transport for example the stimulation of Cl^-^ (Czerucka and Rampal, 2002; Borthakur et al., 2008) and Na^+^ (Singh et al., 2012) exchangers in Caco-2 cells. However, few studies were realized *in vivo* (Girard et al., 2005a,b; Singh et al., 2012) and little is known about cellular mechanisms.

The objective of the present study is to investigate *in vivo* the antidiarrheal effects of two selected probiotic strains, *Bacillus subtilis* CU1 or *Lactobacillus plantarum* CNCM I-4547 using two experimental diarrhea models [castor oil or bacterial lipopolysaccharide (LPS) induced]. Colonic paracellular permeability and short-circuit current were measured in Ussing chambers. Expression in the colonic mucosa of two important intestinal ion transporters, the CFTR and the Na^+^/H^+^ exchanger 3 (NHE3) as well as the Toll Like receptor-4 (TLR-4) were assessed to investigate the underlying mechanism of antidiarrhea effect of probiotics.

## Materials and Methods

### Animals

Male DBA/2 (18–20 g body weight) and NMRI (25–30 g) mice (Janvier, Le Genest St Isle, France) were housed in propylene cages kept in a temperature controlled room (21°C). They were allowed free access to water and fed *ad libitum* (UAR pellets; Epinay, France). Experimental protocols were approved by the Local Institutional Animal Care and Use Committee. The Toxalim animal facility (INRA, UMR 1331, Toulouse, France) is licensed by the French Ministry of Agriculture (agreement B31.555.13). All animal experiments complied with the European Union regulation, as reviewed by the regional ethics committee (CNREEA; MP/03/62/11/11).

### Bacterial Strains and Treatment

*Bacillus subtilis* CU1 (LifeinU^TM^ *Bacillus subtilis* CU1) (CNCM I-2745) and *Lactobacillus plantarum* CNCM I-4547 were produced and provided by Lesaffre (Lesaffre, France). A suspension in physiological saline solution (NaCl 0.9%) was prepared each day with lyophilized bacteria. Mice were orally gavaged with saline solution (controls) or *B. subtilis* CU1 or *L. plantarum* CNCM I-4547 at a dose of 10^9^ CFU/day for 14 days.

### Fecal Water Excretion Assessment *in Vivo*

Mice received the probiotic (*B. subtilis* CU1, *L. plantarum* CNCM I-4547) or the saline solution (control) treatment (*n* = 8 each). Diarrhea was induced 24 h after the last administration of probiotic or saline solution. As previously described (Theodorou et al., 2002; Girard et al., 2003), diarrhea was induced in DBA/2 mice by intravenous administration of *Salmonella enteriditis* LPS (Sigma-Aldrich, Saint Quentin Fallavier, France) at a dose of 15 mg/kg, or intragastric administration (0.2 ml) of castor oil (Sigma) in NMRI mice. Mice were placed in individual cages with the bottom covered with aluminum foil, this allows fecal collection every 30 min during 120 min after LPS or castor oil administration. Each pool of fecal samples was weighed, heated at 100°C for 24 h, and weighed again. The difference between wet and dry matter corresponding to water excretion was used to evaluate diarrhea after LPS or castor oil.

To compare the efficacy of probiotic treatment, antidiarrheal reference loperamide (Sigma-Aldrich) was given orally, 1 h before LPS or castor oil administration, at a dose of 1 mg/kg.

### Colonic Paracellular Permeability and Short Circuit Assessment *ex Vivo*

Colonic paracellular permeability and short circuit current were measured using Ussing chamber. Mice received the probiotic (*B. subtilis* CU1, *L. plantarum* CNCM I-4547) or the saline solution (control) treatment (*n* = 6 each). Diarrhea was induced by LPS in DBA/2 mice or castor oil in NMRI mice. Mice were sacrificed 1 h after saline solution, LPS or castor oil administration. Portions of proximal colon (exposed area, 0.5 cm^2^) were mounted into Ussing chambers (Physiological Instruments, San Diego, CA, United States), each side containing 5 ml of Krebs buffer gassed with 95% O_2_/5% CO_2_. Measurements were taken after a 20-min equilibration period. Paracellular permeability was assessed by measuring mucosal-to-serosal flux of fluorescein isothiocyanate (FITC)-labeled 4 kDa dextran (Sigma). FITC-dextran was added to the mucosal side [2.2 mg/ml, final concentration) and fluorescence was measured at the serosal side after 60 min. Results were expressed as the flux of dextran crossing the epithelial barrier (nmol/h/cm^2^)].

Short-circuit current (Isc) was continuously monitored following a 20 min equilibration period. Isc, expressed as μA/cm^2^, was an indicator of ion exchange across colonic membrane.

### Occludin Expression Assessment *ex Vivo*

Occludin expression on extracted colonic tissue was evaluated using Western blot method. DBA/2 mice received the probiotic (*B. subtilis* CU1, *L. plantarum* CNCM I-4547) or the saline solution (control) treatment for 14 days (*n* = 6 each). Proteins from pieces of the proximal colon were extracted with RIPA buffer (1% Ipegal, 0.5% deoxycholic acid, and 0.1% sodium dodecyl sulfate in Tris-buffered saline 1X; pH 7.4) diluted with protease inhibitor cocktail (Roche Diagnostics, Mannheim, Germany). Clear lysates were prepared by centrifugation at 10,000 × *g* for 10 min, and protein concentrations were assessed using the BC Assay Uptima kit (Interchim, Montluçon, France). Equal amounts of each extract were separated by SDS/PAGE and transferred onto nitrocellulose membranes (Whatman, Dominique Deutscher, Brumath, France). Membranes were blocked with Odyssey blocking buffer (Rockland, Tebu-bio, France) for 1 h at room temperature, and then incubated overnight at 4°C with primary antibodies. Immunoblotting was performed using polyclonal rabbit anti-occludin antibodies (Zymed, Cergy Pontoise, France) diluted 1/500 in Odyssey blocking buffer. After washing in PBS-Tween, membranes were incubated for 1 h at room temperature with fluorescent CF770 anti-rabbit antibodies (Biotium, Hayward, CA, United States) diluted 1/20,000 in Odyssey, and rewashed in PBS-Tween. Membranes were incubated with internal GAPDH (Cell Signaling Technology, Ozyme, Saint Quentin-en-Yvelines, France) 1 h at room temperature, diluted 1/1000 in Odyssey blocking buffer, and then 1 h with secondary antibodies after washing. Membranes were scanned and band intensity was analyzed on infrared imaging system Odyssey (Li-Cor, Lincoln, NE, United States). Occludin expression was assessed relative to GAPDH for each analyzed sample.

### CFTR, NHE3 and TLR4 Protein Expression Assessment *ex Vivo*

CFTR, NHE3, and TLR4 protein expression on membrane of colonocytes was determined by immunohistochemistry methods. DBA/2 mice received the probiotic (*B. subtilis* CU1, *L. plantarum* CNCM I-4547) or the saline solution (control) treatment (*n* = 6 each) for 14 days. At day 15, mice were sacrificed and specimens of proximal colon (1 cm long) were removed and washed with NaCl 0.9%, fixed in 4% buffered paraformaldehyde (6 h) and immersed for 24 h in 30% sucrose at 4°C. Samples were embedded in Neg 50 medium (Microm, Francheville, France) and frozen in isopentane at -45°C. Cryostat sections were post-fixed with acetone (10 min, -20°C) and hydrated in phosphate-buffered saline (PBS). After incubation in blocking solution (PBS containing 1% bovine serum albumin (BSA) and 2% donkey serum), sections were incubated overnight at 4°C with rabbit polyclonal antibodies anti-CFTR (1/100, Abcam, Paris, France), anti-NHE3 (1/200, CliniSciences, Nanterre, France) or anti-TLR4 (1/200, Abcam). Sections were then washed in PBS and incubated for 1 h at room temperature with Alexafluor 488-conjugated IgG donkey anti-rabbit (1/2000, Life Technologies, Saint-Aubin, France). Sections were mounted in Prolong gold antifade mounting medium with DAPI (Life Technologies) and examined under a Nikon 90i fluorescence microscope (Nikon, Champigny-sur-Marne, France). Immunofluorescence analysis was performed in a blinded fashion on five fields for each mouse (*n* = 6 per group) with 60X immersion objective. For each field, a phase contrast picture for locating the tissue structure and a fluorescence picture for quantification were taken. An area of interest corresponding to the epithelium apical region (CFTR and NH3) or to the entire epithelial cells (TLR4) has been selected (Nébot-Vivinus et al., 2014). Areas (μm^2^) and fluorescence intensities were measured employing the software Nis-elements Ar (Nikon). Results were expressed in total fluorescence intensity per square micrometer of epithelium.

### Statistical Analysis

For each parameter studied, data were expressed as mean (± SEM). For statistical analysis, Prism 4.0 (GraphPad, San Diego, CA, United States) was used. Comparisons between groups were performed by non-parametric unpaired Student’s *t-*test and by analysis of variance (one-way ANOVA) followed by a Bonferroni’s post-test. A value of *P* < 0.05 was considered statistically significant.

## Results

### Fecal Water Excretion *in Vivo*

Efficacy of oral administration of *B. subtilis* CU1 or *L. plantarum* CNCM I-4547 were assessed in mice model of diarrhea induced by castor oil or LPS, in comparison to control mice (receiving saline solution).

**FIGURE 1:**
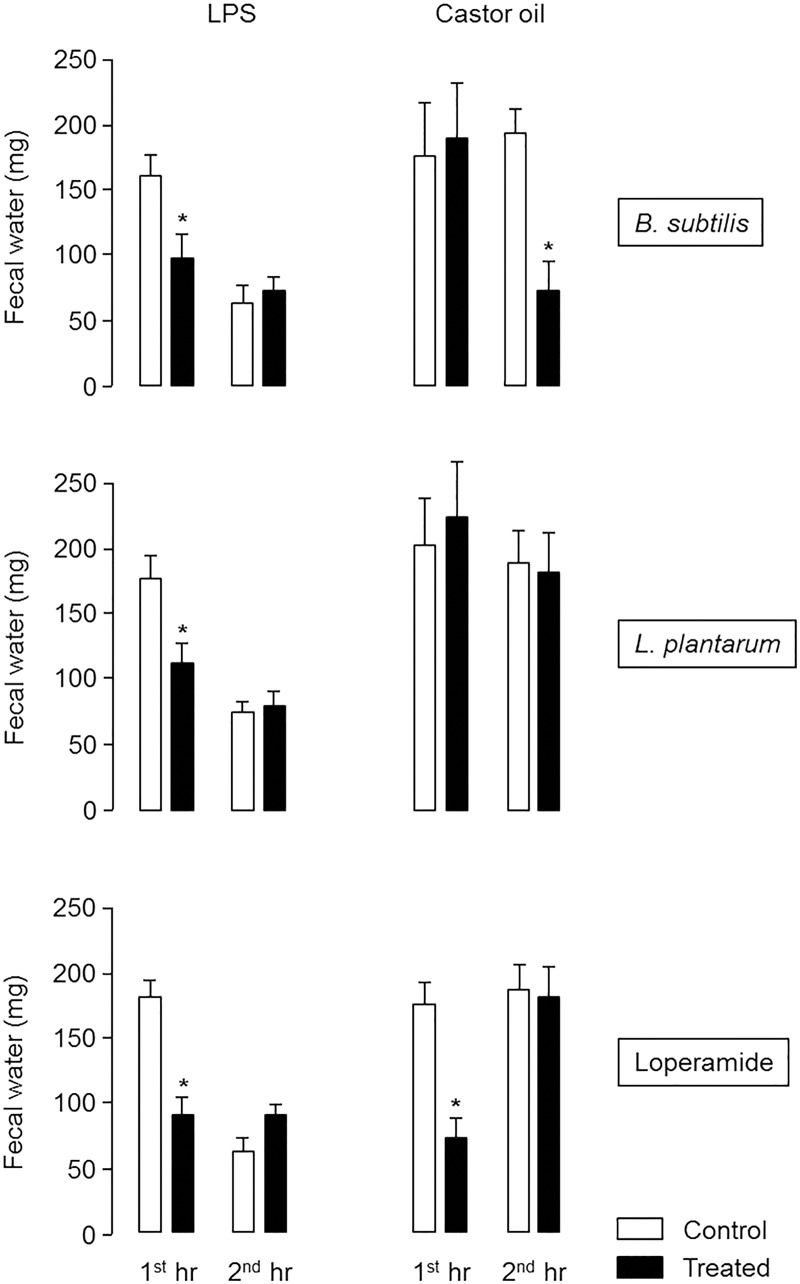
Fecal water excretion during the first and second hour after LPS or castor oil administration in control mice and mice daily treated for 2 weeks by *B. subtilis* CU1 or *L. plantarum* CNCM I-4547, or receiving loperamide orally 1 h before LPS or castor oil. ^∗^*P* < 0.05 vs. control. The results are the average of three independent animal experiences (8 mice in each group) and two independent animal experiences (10 mice in each group) for LPS and castor oil model respectively.

As represented in **Figure 1**, LPS induced diarrhea mainly during the first hour whereas castor oil induced similar level of diarrhea in the first and in the second hour after treatment. In LPS model, *B. subtilis* CU1 reduced water excretion by 37.5% and *L. plantarum* CNCM I-4547 by 39.5% during the first hour in the same range as loperamide (52.0%). In castor oil model, only *B. subtilis* CU1 reduced water excretion by 54.5% during the second hour after castor oil administration and loperamide by 60.5% during the first hour (**Figure 1**). No impact on water excretion was observed with *L. plantarum* CNCM I-4547 in the castor oil model.

### Colonic Paracellular Permeability (CPP) *ex Vivo*

Colonic paracellular permeability was studied by measuring the permeability to dextran in mice colonic tissues mounted in Ussing chambers, 1 h after oral challenge with saline, LPS or castor oil. The flux of dextran was significantly increased by 180% in LPS administered mice and by 66% in those administered with castor oil compared to control mice.

**FIGURE 2:**
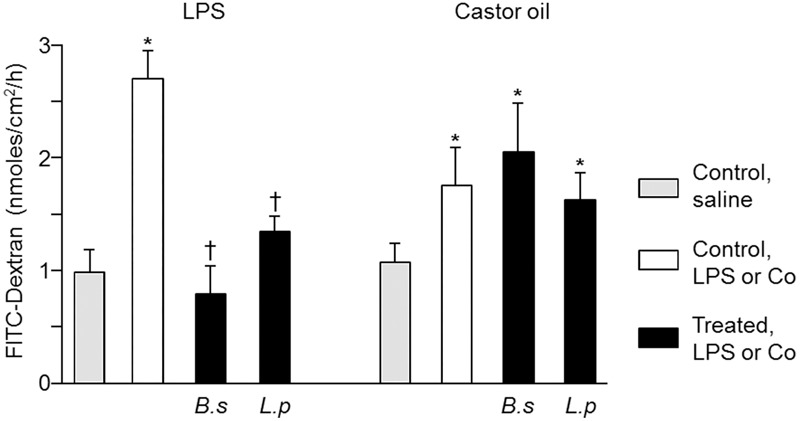
Colonic paracellular permeability after 2 weeks of treatment with *B. subtilis* CU1 (*B.s*), *L. plantarum* CNCM I-4547 (*L.p*) or saline (control). FITC-Dextran flux through the colonic mucosa mounted in Ussing chamber, 1 h after LPS or castor oil (^∗^*P* < 0.05 vs. control, saline; ^†^*P* < 0.05 vs. control LPS or castor oil). The results are the average of two independent animal experiences (6 mice in each group).

The CPP increase induced by LPS was abolished in mice pre-treated with *B. subtilis* CU1 or *L. plantarum* CNCM I-4547. The CPP increase induced by castor oil was not significantly reduced in probiotics administered mice (**Figure 2**).

### Short-Circuit Current Monitoring *ex Vivo*

Colonic short-circuit current (*I*_sc_) was determined also in Ussing chambers 1 h after oral challenge with saline, LPS or castor oil (**Table 1**). LPS and saline did not modify the *I_sc_* in mice administered with *B. subtilis* CU1 or *L. plantarum* CNCM I-4547 (**Table 1**). However *I_sc_* was significantly decreased after castor oil challenge in *B. subtilis* CU1 treated mice (**Table 1**).

**Table 1:** Colonic short-circuit current (μA/cm^2^) determined in Ussing chambers 1 h after oral treatment of saline (0.9% NaCl), LPS (15 mg/kg, i.v) or castor oil (200 μl per os) in mice administered for 2 weeks with saline solution, *B. subtilis* CU1 or *L. plantarum* CNCM I-4547 (10^9^ cfu/day).

Treatment	Diarrheic agent		
	Control	LPS	Castor oil
Saline	111.5 (±10.0)^#§^	103.5 (±5.1)	105.6 (±6.3)
*B. subtilis* CU1	119.3 (±9.2)	103.9 (±9.9)	92.5 (±5.6)^∗^
*L. plantarum* CNCM 1-4547	106.6 (±8.8)	109.1 (±7.9)	115.0 (±8.4)

*^#^Data were expressed as mean (±SEM). ^∗^*P* < 0.05 vs. control. ^§^The results are the average of three independent animal experiences.*

### TLR4, Occludin, CFTR and NHE3 Expression *ex Vivo*

**FIGURE 3:**
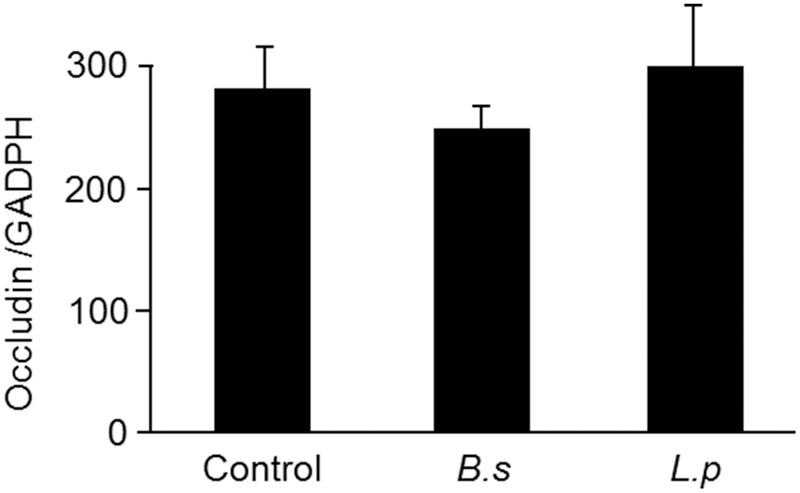
Tight junction protein expression after 2 weeks of treatment with *B. subtilis* CU1 (*B.s*), *L. plantarum* CNCM I-4547 (*L.p*) or saline (control). Occludin expression (Western immunoblotting) in absence of LPS or castor oil administration. The results are the average of two independent animal experiences (6 mice in each group).

To explore possible mechanisms that can be implicated in the antidiarrheal effects observed in our study, we first analyzed the effect of the probiotic strains on the expression of the tight junction protein, occludin. Occludin expression in colonic mucosa was not modified after treatment with either *B. subtilis* CU1 or *L. plantarum* CNCM I-4547 (**Figure 3**).

**FIGURE 4:**
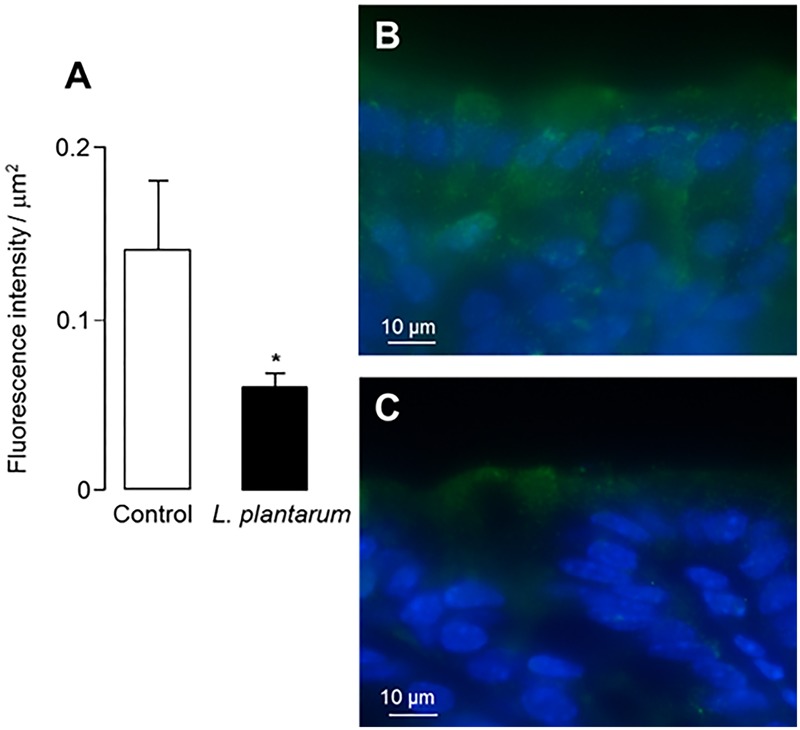
TLR4 immunoreactivity. Intensity of fluorescence associated with TLR4 immunoreactivity in control mice and after treatment for 2 weeks by *L. plantarum* CNCM I-4547 **(A)**. Colonic sections showing TLR4 immunoreactivity (green) in control mice **(B)** and after *L. plantarum* CNCM I-4547 treatment **(C)**. ^∗^*P* < 0.05 vs. control. The results are the average of one animal experience (6 mice in each group).

Then, we assessed the expression of TLR4 at the apical membrane of colonocytes. TLR4 expression was not significantly different after treatment with *B. subtilis* CU1 or *L. plantarum* CNCM I-4547, in comparison to controls. However, TLR4 immunoreactivity determined on the entire colonic mucosa was significantly lower (*p* < 0.05) after *L. plantarum* CNCM I-4547 treatment and remained unchanged after *B. subtilis* CU1 treatment (**Figure 4**).

**FIGURE 5:**
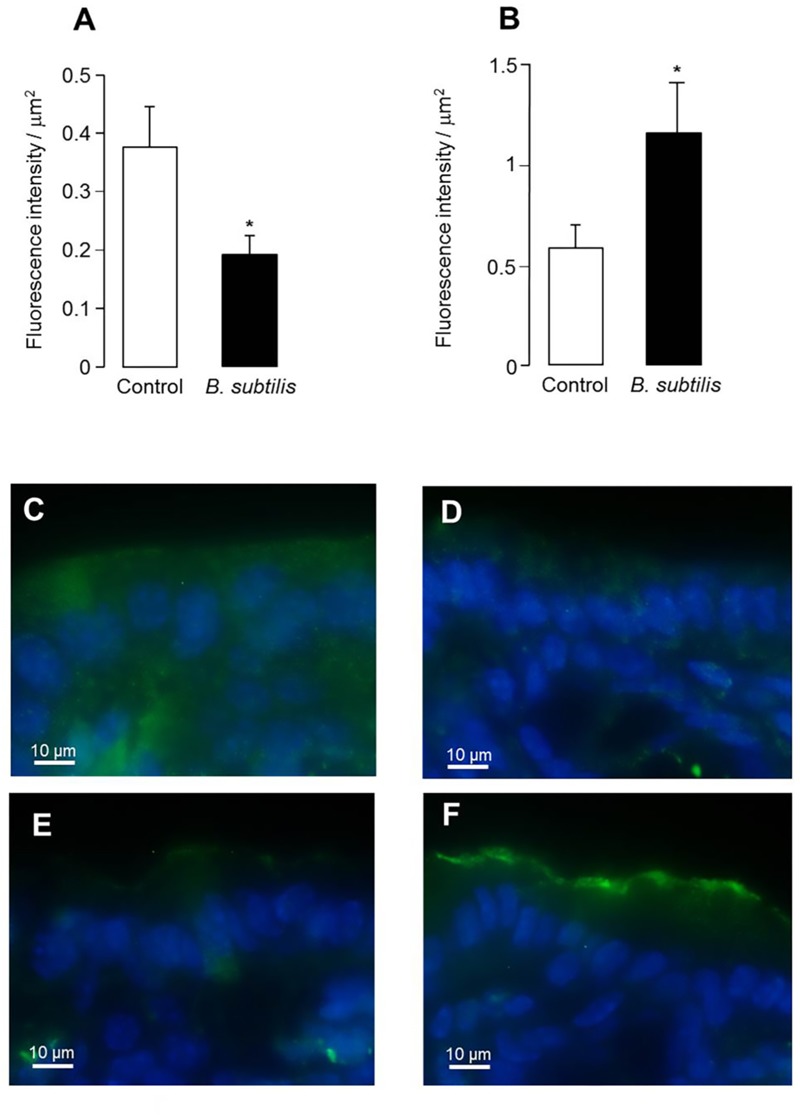
CFTR and NHE3 immunoreactivity. Intensity of fluorescence associated with CFTR **(A)** and NHE3 **(B)** immunoreactivity in control mice and after treatment for 2 weeks by *B. subtilis* CU1. Colonic sections showing CFTR immunoreactivity (green) in control mice **(C)** and after *B. subtilis* CU1 treatment **(D)**. Colonic sections showing NHE3 immunoreactivity (green) in control mice **(E)** and after *B. subtilis* CU1 treatment **(F)**. ^∗^*P* < 0.05 vs. control. The results are the average of one animal experience (6 mice in each group).

Finally, we assessed the expression of two important colonic ion transporters, CFTR and NHE3, at the mice apical surface of colonocytes. CFTR expression was significantly lower in mice treated by *B. subtilis* CU1, compared to control (*p* < 0.05) (**Figure 5**). NHE3 expression was doubled in mice treated by *B. subtilis* CU1, compared to control (*p* < 0.05) (**Figure 5**). Treatment with *L. plantarum* CNCM I-4547 did not impact CFTR and NHE3 expression levels (data not shown).

## Discussion

The interest of probiotics in the management of diarrhea increases but the knowledge about the mechanisms involved is limited. Results obtained in the animal diarrhea models (castor oil or LPS induced) used in our study pointed out the possibility of using *B. subtilis* CU1 and *L. plantarum* CNCM I-4547 strains in the prophylactic treatment of diarrhea symptom. Moreover, we investigated some of the mechanisms that could be involved in the antidiarrheal actions observed.

In our study, we tested the action of two probiotic strains in LPS-induced diarrhea. Despite the use of this model to test different plant extracts or drugs, to our knowledge no probiotic strain has been tested in this model. The early immune response to systemic LPS administration causes shedding of intestinal epithelial cells leading to a fluid secretion into the intestinal lumen (Williams et al., 2013). In this model, we observed that *B. subtilis* CU1 and *L. plantarum* CNCM I-4547 may have a preventive effect against LPS induced diarrhea in mice.

Mice supplementation with *B. subtilis* CU1 or *L. plantarum* CNCM I-4547 abolished the CPP increase induced by LPS. However, probiotics were not effective in reducing CPP induced by castor oil.

Lipopolysaccharide is a pathogen-associated molecular pattern (PAMP) recognized by TLR4 (Beutler et al., 2001) which triggers an inflammatory response resulting in an increase of permeability (Williams et al., 2013). As probiotics supplementation improved LPS-induced diarrhea, we studied the expression of occludin and TLR4 in mice supplemented with the probiotics. No impact of any probiotic supplementations was observed in our study on occludin expression. Nevertheless, *L. plantarum* CNCM I-4547 was able to reduce colonic expression of TLR4. Although other mechanisms cannot be excluded, prevention of diarrhea and paracellular permeability by *L. plantarum* CNCM I-4547 in the LPS model might be closely related to TLR4 expression, preventing the posterior signaling pathways. TLR4 signaling and regulation pathways constitute a complex network. Prophylactic treatment of *L. plantarum* CNCM I-4547 decreased intestinal protein levels of TLR4 which would imply negative regulatory mechanisms as degradation of TLR4, down-regulation at transcriptional level or post-transcriptional repression by microRNAs (Villena et al., 2014). Unfortunately, little is known about the involvement of probiotics in these mechanisms.

In a previous study we have found that *L. plantarum* secreted a rich serine-threonine protein, one of the major extracellular proteins produced by such species (Sánchez et al., 2009). This protein released an internal peptide (STp) when cleaved by intestinal proteases. We demonstrated the anti-inflammatory properties of STp and interestingly found that STp may reduce the expression of TLR4 (Bernardo et al., 2012; Al-Hassi et al., 2014). Further studies would be of interest to investigate whether STp could be involved in the antidiarrheal properties of *L. plantarum* CNCM I-4547.

Castor oil induces intestinal mucosa irritation, inflammation and prevents fluid and electrolytes absorption leading to diarrhea (Holowacz et al., 2016). Few probiotics have been tested using this model. Girard et al. (2005a), observed an antidiarrheal effect of *Saccharomyces boulardii* attributed to its impact on water and electrolyte secretion. Holowacz et al. (2016) showed antidiarrheal effect of a bacterial probiotic mixture through the combination of antimotility and antisecretory properties. Interestingly, *B. subtilis* CU1 administration displayed preventive effect on castor oil-induced diarrhea resulting in a reduction of water excretion. In addition, Isc reduction observed in Ussing chamber indicated the impact of *B. subtilis* CU1 on ion transport across colonic mucosa leading us to postulate the involvement of ionic exchangers.

Previously a mixture of *L. helveticus* and *L. rhamnosus* has been shown to reduce or abolish the ileal or colonic increase in short-circuit current observed after an acute stress or a maternal separation in rats (Zareie et al., 2006; Gareau et al., 2007). Similarly, *Bifidobacterium breve* C50 reduced the increase in short-circuit current induced by carbachol or forskolin in the HT29 cell line (Heuvelin et al., 2010). Nevertheless, these studies did not attempt to correlate the effect of the probiotics on short-circuit current with an antidiarrheal action. Furthermore we selected NHE3 which is expressed on the luminal side of colonic epithelial cells and provides a large contribution to Na^+^ absorption (Rajendran and Binder, 1990). The absence of NHE3 in mutant mice results in severe and chronic diarrhea, thereby suggesting that this exchanger may play a significant absorptive role in mouse intestine (Schultheis et al., 1998). In diarrhea, as those induced by *C. difficile* or rotavirus, the expression of NHE3 is inhibited (Halaihel et al., 2000; Hayashi et al., 2004; Greenberg and Estes, 2009; Engevik et al., 2015). In this context, probiotics that contribute to NHE3 upregulation could be useful in the co-treatment of diarrhea. Among the secretory channels, we selected the CFTR channel which plays a major role in Cl^-^ secretion (Greger, 2000). In severe diarrhea induced by some enteropathogens as *Vibrio cholerae*, CFTR expression can be activated by causing ion and fluid secretion in the intestine (Barrett and Keely, 2000). In this context, probiotics that can modulate active ion secretion in the intestinal epithelia, such as *B. subtilis* CU1 can be interesting.

Direct impact of probiotics or their secreted compounds on ionic transport and/or in the expression of CFTR and NHE3 has been previously studied *in vitro* (Czerucka and Rampal, 2002; Borthakur et al., 2008; Heuvelin et al., 2010). However, studies of cellular pathways involved are very brief. Concerning *in vivo* studies, Singh et al. (2012) showed an increase in NHE3 mRNA and protein expression in the colon of mice treated with *L. acidophilus* but mechanisms were not studied.

Interestingly, new highlights in the mode of action of some probiotics includes their capacity to modulate intestinal microbiota. In this context, *in vivo* effects observed in our study can be indirect via the microbiota and its metabolites. Short-chain fatty acids (SCFA) specially butyrate, may stimulate electroneutral Na^+^ uptake mediated by apical NHE3 (Musch et al., 2001) and also mediates inhibition of CFTR Cl^-^ secretion (Vidyasagar and Ramakrishna, 2002). Antidiarrheal effects observed with *B. subtilis* CNCM I-2745, including the modulation of NHE3 and CFTR expression, could be the result of an indirect mechanism on intestinal microbiota. The ability of BSCU1 to increase NHE3 and decrease CFTR expression provides new insight on the mechanism of probiotics on diarrhea.

## Conclusion

Our results indicate that both *B. subtilis* CU1 and *L. plantarum* CNCM I-4547 prophylactic treatments displayed potent antidiarrheal activity. *L. plantarum* CNCM I-4547 can prevent LPS-induced diarrhea possibly through a down-regulation of TLR4 expression. *B. subtilis* CU1 was active against distinct experimental diarrhea, castor oil or LPS, and might increase the capacity of the colon to absorb water in diarrheic conditions through an up-regulation of NHE3 expression. Moreover *B. subtilis* CU1, may decrease intestinal hypersecretion, by down regulating CFTR in the colon.

## Author Contributions

JF, MU, and ML conceived and designed the study, participated in data interpretation, and drafted the manuscript. GL performed the mice experiments and CC performed the immunofluorescence assays. BR contributed to the draft of the manuscript.

## Conflict of Interest Statement

ML holds a full time position with ENNOLYS, a LESAFFRE Group subsidiary. BR holds a full time position with LESAFFRE HUMAN CARE, a LESAFFRE Group subsidiary. The remaining authors declare that the research was conducted in the absence of any commercial or financial relationships that could be construed as a potential conflict of interest.

## Abbreviations

CFTR: cystic fibrosis transmembrane conductance regulator
CPP: colonic paracellular permeability
LPS: lipopolysaccharide
NHE3: sodium hydrogen exchanger 3
TLR4: toll-like receptor 4
